# Children’s Mental Health in the Time of COVID-19: How Things Stand and the Aftermath

**DOI:** 10.21315/mjms2020.27.5.15

**Published:** 2020-10-27

**Authors:** Mohammad Hilal Atthariq Ramadhan, Ayu Kartika Putri, Diana Melinda, Umrohtul Habibah, Utami Nurul Fajriyah, Syarifah Aini, Bintang Arroyantri Prananjaya, Diyaz Syauqi Ikhsan

**Affiliations:** 1 Faculty of Medicine, Universitas Sriwijaya, Palembang, Indonesia; 2 Department of Psychiatry, Faculty of Medicine, Universitas Sriwijaya, Palembang, Indonesia

**Keywords:** children, mental health, coronavirus, pandemic effects

## Abstract

Currently, the world is facing immediate and unexpected changes every day due to the coronavirus disease 2019 (COVID-19) pandemic. These environmental stimulations have an impact on mental health, particularly in the case of children. Worldwide measures such as quarantine, social and physical distancing, and school closure can potentially take a toll on children’s mental health, in both the short and long terms. Grief, adjustment disorder and acute stress disorder (ASD) are some of the mental health issues that children are prone to suffer during a pandemic, leading to mood disorder, psychosis and even suicidal tendency, if not addressed and treated timely. As the pandemic continues, it is crucial to monitor children’s mental health status. Parents/caregivers must receive continuous guidance on handling the situation appropriately. Both professionals and families/caregivers must tend to children’s mental health needs to safeguard their overall well-being.

## Introduction

On 31 December 2019, new pneumonia cases with unknown causes were reported in Wuhan, China (1–3). This report led to the discovery of a novel coronavirus in January 2020, which was named coronavirus disease 2019 (COVID-19) (2). Initially, this disease was believed to be transmitted from animals to humans before further findings indicated human-to-human transmission (3). Human-to-human transmission is suspected to occur upon close contact with an infected person via their respiratory droplets when they sneeze, cough or talk (3). Despite the announcement of COVID-19 as a Public Health Emergency of International Concern in January 2020, little action was taken until the virus rampage worsened and infected millions of people worldwide (1, 4). Considering how COVID-19 spread like wildfire across continents, the World Health Organization (WHO) finally declared it as a pandemic in March 2020 (1, 4).

Ever since COVID-19 has been declared as a global pandemic (1, 4), information related to its symptoms, death toll and precautions has become a part of everyday news. This has certainly created awareness amongst people about the disease and its impact on our physical health, but the implications on mental health have been overlooked. Mental health is vital for our overall well-being, and especially for children as their cognitive and mental abilities are in the developmental stage.

Studies show that the mental health of a person depends on the interaction between the genes and the environment of that person (5). The genetic make-up of a person serves as a blueprint of mental health that can be modified in both positive and negative ways based on environmental stimulation (5). For example, disruption of routine, such as the one observed during the COVID-19 pandemic, can be considered as a change in the environment. Social distancing, school closures and staying at home for an uncertain period can take a toll on children’s mental health. Further studies and reviews are warranted to evaluate the short- and long-term effects of the current situation on children’s mental health to prevent and reduce associated risks. This review aims to report on the current knowledge about children’s mental health development and well-being worldwide.

## The COVID-19 Pandemic and Children’s Mental Health

Mental health care is significant for children (4). The onset of most mental health issues is observed in childhood, making it imperative that any related signs identified during this period need to be recognised early and treated appropriately. Otherwise, such issues can prompt negative health and social outcomes in the lives of the children, both in the present and the future.

Children comprise a vulnerable population in society and require constant care and guidance. They need company and assistance, making the role of parents and/or guardians pivotal during this time. With so many radical changes taking place worldwide, children’s mental health can be impacted both in the short and long terms. Irrespective of whether they live with or without family and/or caregivers, children’s mental well-being is bound to be affected by the quarantine policy (6).

## Impact of the COVID-19 Pandemic on Children’s Present Mental Health

There were only some studies discussing the impact of a pandemic on the mental health of children (7–9). However, a study on 586 parents who experienced the H1N1 pandemic reported that children’s mental health was affected significantly by the pandemic (7).

Children who stayed with their families during a disaster and/or outbreaks experienced stress to some degree due to the environmental changes they faced (8). It also appears that children who stayed at home during the pandemic were prone to experience adjustment disorder, acute stress disorder (ASD) and grief (8). These findings are in line with the cause behind ASD, which is the most commonly identified psychiatric disorder following a traumatic event. Grief can also be anticipated both during and after a pandemic, which becomes worse after losing loved ones (10). It can be further complicated by maladaptive thoughts, feelings and behaviours, such as avoiding and/or withdrawing from social interactions (10).

Children with ASD may also experience sleeping problems, with or without nightmares that are related or unrelated to the traumatic event (5). This is why parents/caretakers must be alert when their children show or express any difficulties in sleeping and/or are having nightmares during the pandemic. Their recreational and studying abilities can be affected as well, since they may have trouble concentrating, in addition to becoming irritable, fearful, distractible, anxious or even depressed. Children may avoid situations and discussions relating to or reminding of the trauma. They may start withdrawing themselves from the external world as they shun everyone around and continue to feel numb from the aforementioned mental health issues (5).

## Impact of the COVID-19 Pandemic on Children’s Future Mental Health

It has also been noted that a significant number of children who were quarantined were prone to experience post-traumatic stress disorder (PTSD) (8, 9). The pandemic and the drastic changes caused by it can be considered as traumatic events for some children. Children with PTSD often exhibit symptoms like having trouble sleeping and may have nightmares related to the event (5). Moreover, children get easily irritated with little or no provocation and start to feel detached from their surroundings (5). They may exhibit avoidance and numbness to requests regarding discussions or recollections related to the traumatic event (5). Some are even unable to recall the event, which is a condition known as dissociative amnesia (5).

PTSD among children in the general population is predicted to be around 8%–9% (5). A study conducted by Sprang in 2013 reported that children in isolation and quarantine have PTSD rates similar to those who are exposed to the disaster, and it is known that children who are exposed to the disaster have a higher frequency of PTSD, which could range from 5%–95% (8). PTSD is a condition that must be anticipated and possibly prevented both during and after a pandemic.

However, the rising stress among children living with their families can be tamed to some extent (11). The additional focus needs to be given to children who are being quarantined away from their parents/guardians, whose family is dysfunctional and who have witnessed ongoing abuse issues in their household. The latter circumstances give rise to a higher possibility of having mental health problems. Separation is known to put children at the risk of suffering from psychiatric disorders (11). Losing parents and/or living separately from parents during childhood can harm children’s future mental health, making them more susceptible to mood disorder, psychosis and committing suicide (12, 13).

## Recent Studies Regarding Children’s Mental Health During the COVID-19 Pandemic

A preliminary study was conducted in China with 320 children (aged 3–18 years old) who were quarantined owing to the COVID-19 pandemic. The study aimed to screen psychological and behavioural problems among these children during their quarantine. It was identified that clinginess, distraction, irritability and fear of asking questions about the pandemic were common among these children (14). Based on the aforesaid study, it was found that children among different age groups showed slightly different symptoms. Younger children (3–6 years old) were more clingy and more scared about their family members contracting the virus (14). Older children (6–18 years old) were more inattentive or persistently inquisitive (14).

Another study conducted with 2,230 elementary students quarantined for 3–4 months in China reported that 22.6% of them exhibited depressive symptoms, 18.9% demonstrated anxiety symptoms and 37.3% were worried about contracting the virus (15). These studies illustrate how a pandemic may influence children’s mental health.

## School Closure and Its Effects on Children’s Mental Health

School closure due to the COVID-19 pandemic is also impacting children’s mental health. Apart from education, schools provide health interventions (for both physical and mental well-being) that can be easily accessed by the students when on campus (16, 17). With schools shut down in as many as 188 countries since April 2020, it is estimated that more than 90% of students are now staying at home (18). This situation demands a special need to focus on the psychological well-being of these students because such suspensions deprive them of access to assets that they typically have in schools (17).

A study conducted in the United Kingdom reported that the majority of young people with a history of mental illness stated that the pandemic had exacerbated their conditions; one-fourth of this group expressed that they could not receive their usual mental health care (8). School schedules act as lifelines for students facing mental health issues by offering them a significant coping mechanism (17). Another downside to school closure is its adverse impact on children requiring special education. These children will suffer more as their daily routine is altered. As a result, they can easily get irritated and frustrated (18).

## Stressors During and After the Pandemic

The COVID-19 pandemic can give rise to parenting stress as well, since both parents/caretakers and children are living with stress and in fear (18). Moreover, the overwhelming news from media can be upsetting for both parents and children as it adds to the stress. Therefore, quarantine can have a negative emotional impact on everyone. The effects may vary from stress, irritability, sleeping problems, fear, confusion, anger, frustration, boredom and depression, some of which may persist after the quarantine (19, 20). Longer quarantine duration, inadequate basic supplies (accommodation, food, water and clothes) and inability to access medical services are some of the stressors that can worsen the emotional outcomes (9, 19, 20). On the other hand, financial problems and stigma are the stressors commonly known to influence post-quarantine life (9).

## Talking to Children

It must be noted that children’s emotional status is in sync with that of the adults close to them (21). If parents/caretakers behave unpredictably and unexpectedly, children see it as a threat and tend to get anxious (21).

A study shows that children as young as 2 years old can sense changes around them; they notice the absence of their caretakers and consequently become restless (21). Therefore, parents/caretakers must talk to their children and try to understand how they feel about the pandemic situation (19, 21).

If parents/caretakers do not discuss the changes happening in their families with their children, the children will try to figure it out themselves (21). Without a clear explanation from the adults around them, children may experience negative emotional responses such as anxiety and guilt. Therefore, parents/caretakers need to be honest about the changes happening in their families.

However, it is to be noted that when talking to children, the information which is being shared must be adjusted to suit their level of age and understanding. This can lead to effective communication without underestimating or overestimating the children’s understanding; for example, when talking to children aged 4–7 years old, parents/caretakers have to be careful of their children blaming themselves for causing the illness because of their previous bad behaviour (21). Children at that age are influenced by imaginative thinking and hence believe that their wishes, thoughts or irrelevant actions can create external events (21). For instance, they may believe that their friend is sick because they wished for it. For this reason, listening to children is as important as talking to them.

## Where to Seek Help During the Pandemic

Many institutions and organisations like the WHO, UNICEF, American Psychiatric Association, European Society for Child and Adolescent Psychiatry and several others are guiding parents/caretakers, through their websites, on how to cope with today’s situation and on parenting during the COVID-19 pandemic (22). WHO, for example, provides infographics on its webpage for parents/caretakers on how to interact with their children during the pandemic (23). The advisory includes the followings (23):

- spend time with the children, listen and give them full attention
- stay positive
- create a flexible but consistent daily routine
- avoid bad behaviour
- manage stress, and
- talk to the children about COVID-19

## What Can Be Done During the Pandemic?

Interestingly, challenging times often offer opportunities for dealing with situations creatively and making the best of them. Improving communication and bonding over games, engaging in physical activities with all family members, and singing and reading together can help alleviate the stress, worry, and fear that children may experience (9, 11). Participating in online classes is encouraged; however, the use of social media should be monitored to avoid exposure to overwhelming news (10). Routine and structured activities, new or old, can help children in coping with the quarantine (10).

## Conclusion

After declaring COVID-19 a global pandemic in March 2020, WHO urged all countries to take necessary and stringent measures to reduce the virus’s transmission. Physical distancing, school closure, staying at home and working from home along with local and international travel restrictions have been implemented by many countries. The impact of these measures affects all societies and their members. School closure, physical distancing and staying at home can affect a child’s mental health, in both short and long terms. Studies have stated that children who were staying at home in times of a pandemic were more likely to develop adjustment disorder, ASD, grief, clinginess, distraction, irritability and fear. They are also at a higher risk of suffering from PTSD after the pandemic. Parents/caretakers are being advised to be there for their children by communicating, bonding and performing activities together at home to ease and prevent any psychological issues that children may encounter. As the pandemic continues, it is pivotal to build a connected and resilient community to safeguard children’s mental health; it is equally important to be prepared to provide support and care to the children facing the aftermath of the COVID-19 pandemic.
